# A multi-aspect analysis of two analogous aspergillus spp. belonging to section Flavi: aspergillus flavus and aspergillus oryzae

**DOI:** 10.1186/s12866-023-02813-0

**Published:** 2023-03-15

**Authors:** Waleed Bakry Suleiman

**Affiliations:** Botany and Microbiology Department, Faculty of Science (Boys), Al-Azhar University, The Permanent Camp St., 6th Ward, Nasr City, 11884 Cairo, Egypt

**Keywords:** Analogous strains, *Aspergillus flavus*, *Aspergillus oryzae*, SEM, Secondary metabolites, ITS rDNA

## Abstract

Microfungal isolates were routinely identified depending on both macro and micro morphological characteristics, sometimes, some fungal isolates appeared to be similar and such cases caused severe confusion for mycologists during the preliminary identification. During our previous studies dealing with isolation of fungi for some biotechnological applications; two mystifying species *Aspergillus flavus* and *Aspergillus oryzae* showed similar cultural and macroscopic features. Therefore, the current study aimed to easily distinguish between these two species depending on simple approaches which are routinely followed by a large segment of researchers. Investigation of the macroscopic features was performed to check the fungal growth on four different media (PDA, MEA, YES, and CYA) followed by microscopic examination using an ordinary light microscope, and scanning electron microscope SEM. Also, screening of secondary metabolites for both strains was preliminarily identified to find out the difference between their metabolic profiles. Finally, ITS rDNA was involved to clarify the molecular differences along their partial sequence. Conclusively, the BLAST strategy confirmed the similarity of ITS rDNA segments of both fungal strains that supported our hypothesis. The color of the fungal growth is a very critical factor whereas it is extensively influenced by the type of cultivation media. Accordingly, the YES medium was an inspiring tool assisting in prompt differentiation during the culture investigation step whereas *A. oryzae* and *A. flavus* appeared significant mustard yellow and olive green respectively. During the microscopic examination, the CYA medium also had a robust effect on the formation of the conidial chain whereas the knit long chain was observed in *A. oryzae* while the conidia appeared scattered and not in a chain in the case of *A. flavus*. Likewise, both two strains possessed different metabolic profiles where *A. oryzae* is not an *Afla* toxin producer, unlike *A. flavus*.

## Introduction

Aspergilli are great genera that include more than 180 identified anamorphs, plus the other nine genera with teleomorphs [1]. *Aspergillus* is classified into seven subgenera, which are subsequently subdivided into Sect. [2]. *Aspergillus* classification is complicated and ever-developing as well as the rest genera of other fungi. The identification of genus level is easily performed through recognition of its typical conidiophore, nonetheless, the identification of species level is intricate, for it is classically dependent on different morphological topographies. Cultural characteristics include color, the reverse color of mycelial growth, growth diameter, and secretion of dissolvable pigments and exudates. Microscopic characterization in *Aspergillus* is principally based on the shape and size of the vesicle, conidia, and seriation as well as characteristics of cleistothecia and ascospores. Moreover, all macroscopic and microscopic investigations must be conducted under standardized certain laboratory conditions [3].

*Aspergillus flavus* group was specifically referred to as *Aspergillus* Subgenus *Circumdati* Section *Flavi*, it has interesting aspects due to its industrial applications and toxigenicity. The section *Flavi* was classically subdivided into two groups; the aflatoxigenic species (*A. flavus*, *A. parasiticus*, and *A. nomius*) which give rise to severe agricultural and health problems overall in the universe. The second group comprises the non-aflatoxigenic species (*A. oryzae*, *A. sojae*, and *A. tamarii*) which were traditionally applied in fermented foods production in Asia [4]. Aspergilli could secrete several biologically active chemical compounds such as mycotoxins, immune-suppressants, antibiotics, and cholesterol-lowering agents [5]. Subgenus *circumdati* also could contribute to the industrial field, particularly in biotransformation processes.

Isolation and identification of *A. oryzae* have been reported to be tricky due to the intra-and interspecies variability of isolates and the morphological similarities with *A, flavus* [6]. The distinguishing of *A. flavus* from *A. oryzae* is a noteworthy challenge in all laboratories of mycology, particularly which use the classical techniques depending upon macroscopic and microscopic examination [7]. Some previous research articles [8] deeply studied the differentiation between these two species with highly sophisticated protocols such as (MALDI-TOF MS).

During our previous study dealing with isolation of fungi for the production of oil [9–12] from different agricultural wastes, in addition to screening and biodiversity of fungal communities along the coastline of Alexandria, Egypt [13–15], and screening of some fungi for their ability to produce biologically active enzymes [16], it was considerably noticed that some fungal species appear to be similar to each other in their culture characteristics especially two isolates; *A. flavus* and *A. oryzae*. Hence, this study aimed to find the simplest way to easily distinguish between those two tricky species *A. flavus* and *A. oryzae* through the investigation of their cultural and microscopical features on 4 different media, followed by screening of their secondary metabolic profiles, and finally ITS molecular identification to find out the significant differences. All these strategies were routinely used by a large segment of mycologists.

## Materials and methods

### Fungal strains

The two species *A. flavus* and *A. oryzae* were provided by the unit of culture collection, the Regional Center for Mycology and Biotechnology (RCMB), Al-Azhar University, Cairo-Egypt. They were cultivated onto four different media that are commonly involved in fungal enumeration.

### Cultivation media

#### Potato dextrose agar (PDA) medium

PDA medium (NutriSelect® Basic) composed of potato extract 4 g/L, dextrose 20 g/L, and agar 15 g/L. it was prepared by dissolving 39 g of the ready-made medium, and cooked in one liter of distilled water [17, 18].

#### Malt extract agar (MEA) medium

MEA medium was prepared by suspending 33.6 g of readymade medium (Thermo Fisher Scientific) in one liter of distilled water [19, 20].

#### Yeast extract sucrose agar (YES) medium

YES medium (HIMEDIA, M1797) was prepared by dissolving 40.5 g of the readymade medium into one liter of distilled water [21].

#### Czapek yeast autolysate Agar (CYA) medium

CYA medium (HIMEDIA, M2061) was prepared by dissolving 54.75 g of the readymade medium into one liter of distilled water [22].

All media were sterilized by autoclaving at 1.5 Pa, 121 ^º^C, for 15 min. then poured into Petri dishes for inoculation. Each fungal inoculum [23, 24] was spread regularly onto the agar surface of each medium, incubated at 30 ± 2 ^º^C, and checked daily for suitable fungal growth.

### Investigation of morphological characteristics

#### Culture and microscopic examination

Fungal cultures were submitted for confirmative identification based on their morphological characteristics on malt extract agar medium. The macroscopic features including colony growth, color, texture, and reverse color were reported. Also, the hyphae were checked for their aerial growth on the medium, and the slides were observed under a microscope (400X) [25]. Conidiophores, conidia, vesicles, and branching patterns were identified under a microscope using the identification characteristics key by Klich (2002) [26].

#### Scanning electron microscopy (SEM)

Four-day cultures of both two analogous fungi were submitted to be investigated by SEM, the medium chosen was MEA as the most common growth medium. SEM technique was performed stepwise including fixation by osmium tetraoxide (OsO_4_), followed by tissue processor (Sciences tissue processor model Lynx) for dehydration through immersion in gradual concentrations of ethyl alcohol followed by gradual concentrations of acetone. The third step involved the critical point dryer (EMS 850 apparatus) in which acetone was replaced by carbon dioxide, mounted on stubs to be ready for the gold coating in order to good conductivity by diode gold sputter coater (SPI Module™ Sputter Coater), and finally, the fugal specimen getting ready to be examined by high-vacuum mode of a JEOL JSM-5500LV Scanning Electron Microscope [27, 28].

#### Molecular differences based on ITS rDNA identification

Fungal mycelia were inoculated in yeast extract sucrose broth for 10 days with agitation at 160 rpm, then the fungal pellets were harvested and squashed to a fine powder in a mortar with an appropriate amount of liquid nitrogen, and extraction of DNA was performed using DNeasy kit (Qiagene, Germany) [13], the PCR amplification was carried out in 25 µL as a total volume containing genomic DNA (20 ng), PCR buffer (1X), dNTPs (0.2 mM), Taq DNA polymerase (0.2 U) (Roche Holding AG, Basel, Switzerland), and the primers (10 pmol); ITS1 (5`TCCGTAGGTGAACCTGCGG3`), ITS4 (5`TCCTCCGCTTATTGATATGC3`) [29]. The temperature pattern of the PCR amplification process was conducted as follows: general denaturation step at 94^°^C for 2 min, amplification step for 40 cycles of 60 s at 94^°^C, 90 s at 52^°^C, and 2 min at 72^°^C, and a final extension step at 72^°^C for 10 min.

### Screening of secondary metabolites profiles by thin-layer chromatography

Both two fungal cultures (21-day culture age) were homogenized and macerated in chloroform-methanol 2:1 (v/v) for intracellular and intracellular extraction of secondary metabolites. After filtration, both fungal extracts were subsequently kept at room temperature to allow all solvents to be evaporated, then their dried residues were resuspended in an appropriate volume of methanol and filtered. Thin-layer chromatography (TLC) plate (10 × 10 cm Merck aluminum sheet, silica gel 60, layer thickness 0.2 mm) was loaded with both fungal extracts against Griseofulvin as an authentic reference standard. Chromatographic bands were allowed to be developed by (TEF) toluene - ethyl acetate- formic acid 5:4:1 (v/v/v) as an elution buffer for a while, pursued by multiple ultraviolet scanning at different wavelengths; visible light, long UV_365_, short UV_254_, long UV_365_. 0.5%p-anisaldehyde dissolved in (Conc. H_2_SO_4_ – acetic acid – acetone 5:10:85) was used for spraying the loaded TLC plate which was subsequently heated at (105 °C) in the oven for 10 min. Data of the resulted bands (color, R*f*, and shape) was analyzed according to Paterson & Bridge [30, 31] to primarily predict the chemical constituents of the fungal extracts.

## Results and discussion

### Macroscopic and microscopic features of both fungal strains on different media

**Fig. 1:**
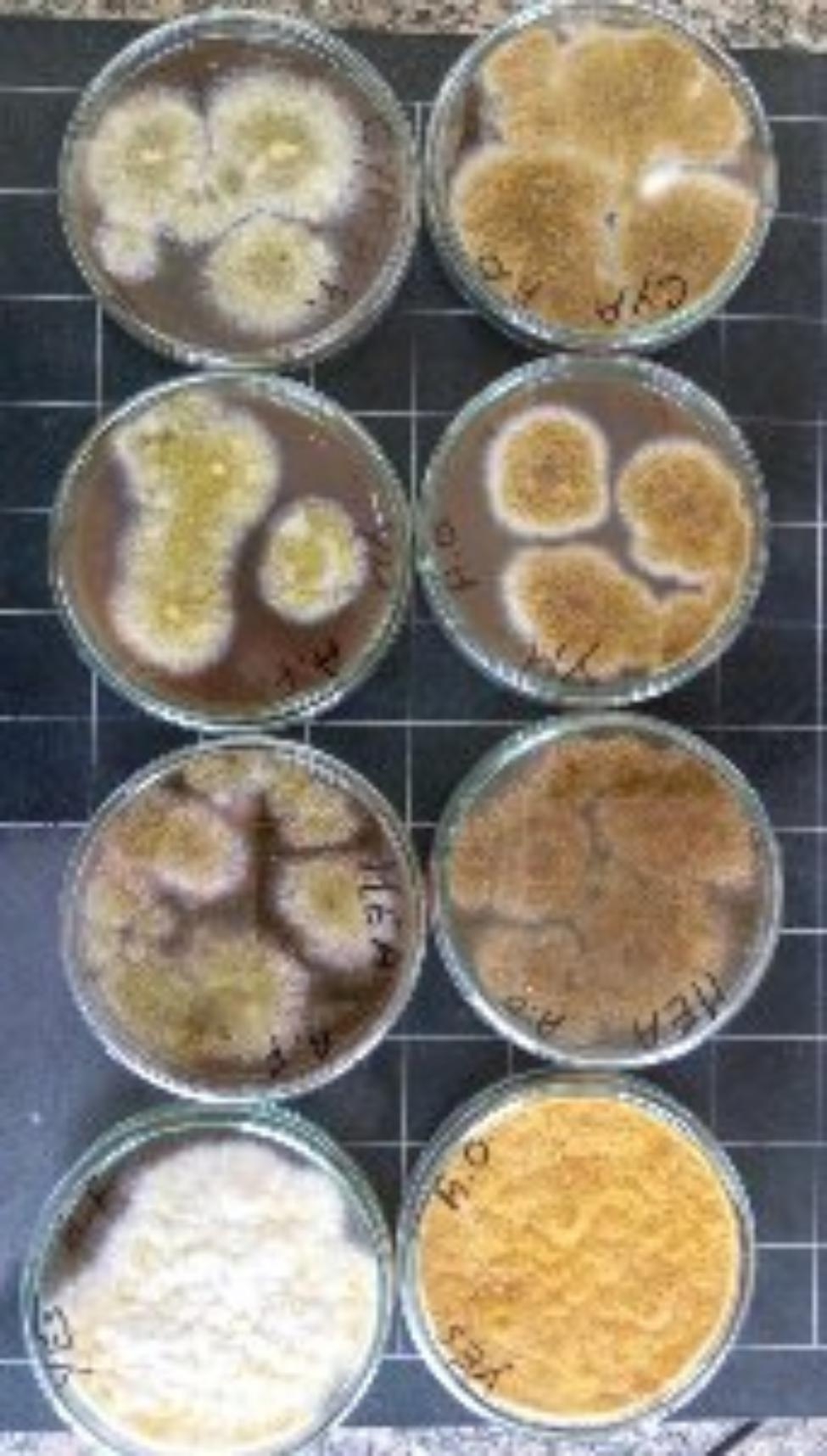
Differences in fungal features were cultivated on four different media for 7 days. The upper row represents the growth of *A. flavus* on YES, MEA, PDA, and CYA (from left to right), and the lower row represents the growth of *A. oryzae* on YES, MEA, PDA, and CYA (from left to right)

Both *A. flavus* and *A. oryzae* were grown on four different cultivation media (PDA, MEA, YES, and CYA) to show the difference in their behavior. Figure (1) exhibited a substantial difference in the culture color which is olive green with white margins in the case of *A. flavus* with condensed growth on YES medium followed by MEA, CYA, and PDA respectively. In the case of *A. oryzae*, the culture color was mustard yellow and the white edges were observed only on CYA, and PDA media while lacking in both YES, and MEA media. The growth of fungal biomass is interestingly affected by the type of medium included for cultivation. Those four media types are commonly used as aspergilli growth media, several studies approved the effect of media types on the growth, sporulation [32], also the growth rate, colony characteristics, and sporulation patterns of 10 fungal isolates that were cultivated on three different media were greatly influenced where lignocellulose agar medium promotes the growth rate for some isolated and the other isolates were promoted by PDA, and CYA media [33].

**Fig. 2:**
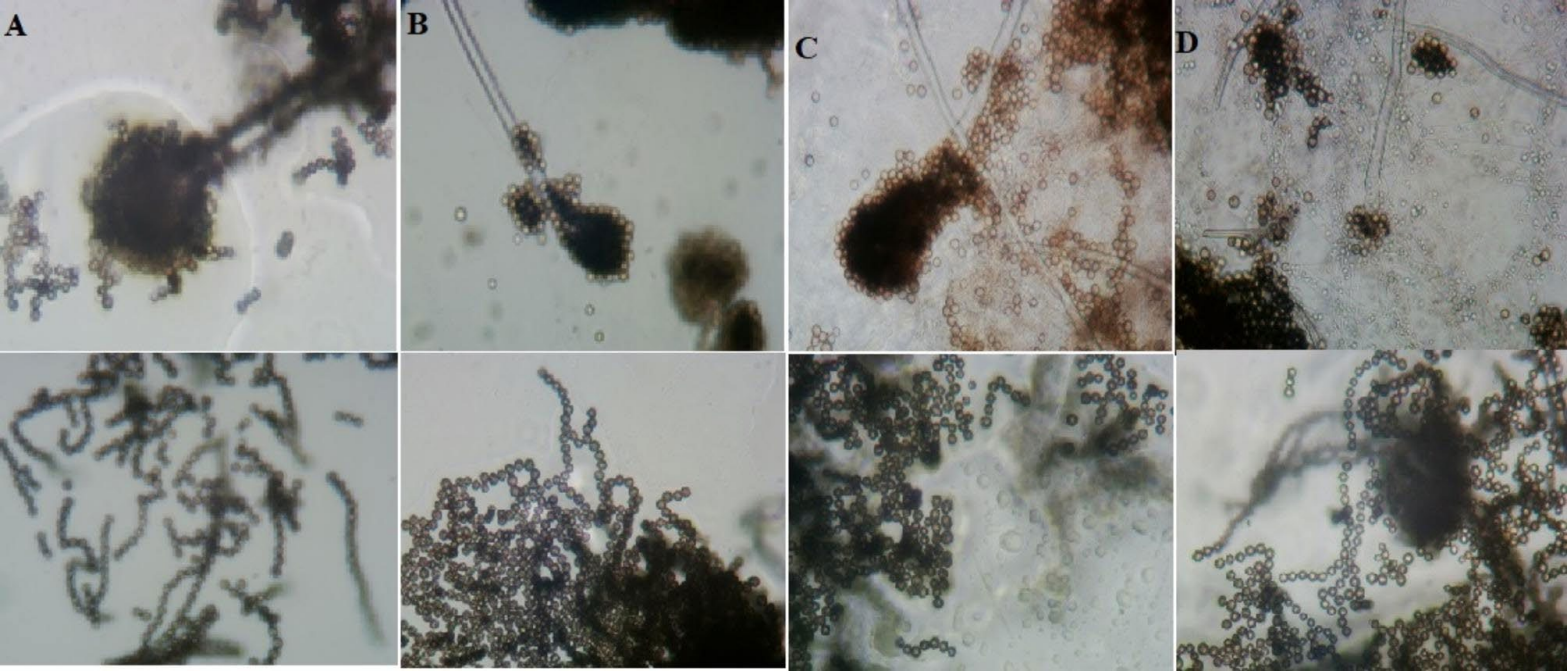
Light microscopy shows the influence of different growth media on the microscopic characteristics of *A. oryzae*. **A**; the vesicle on CYA, and the shape of the conidial chain, **B**; the vesicle on MEA, and the shape of the conidial chain, **C**; the vesicle on PDA, and the shape of conidia chain, **D**; the vesicle on YES, and the shape of the conidial chain

**Fig. 3:**
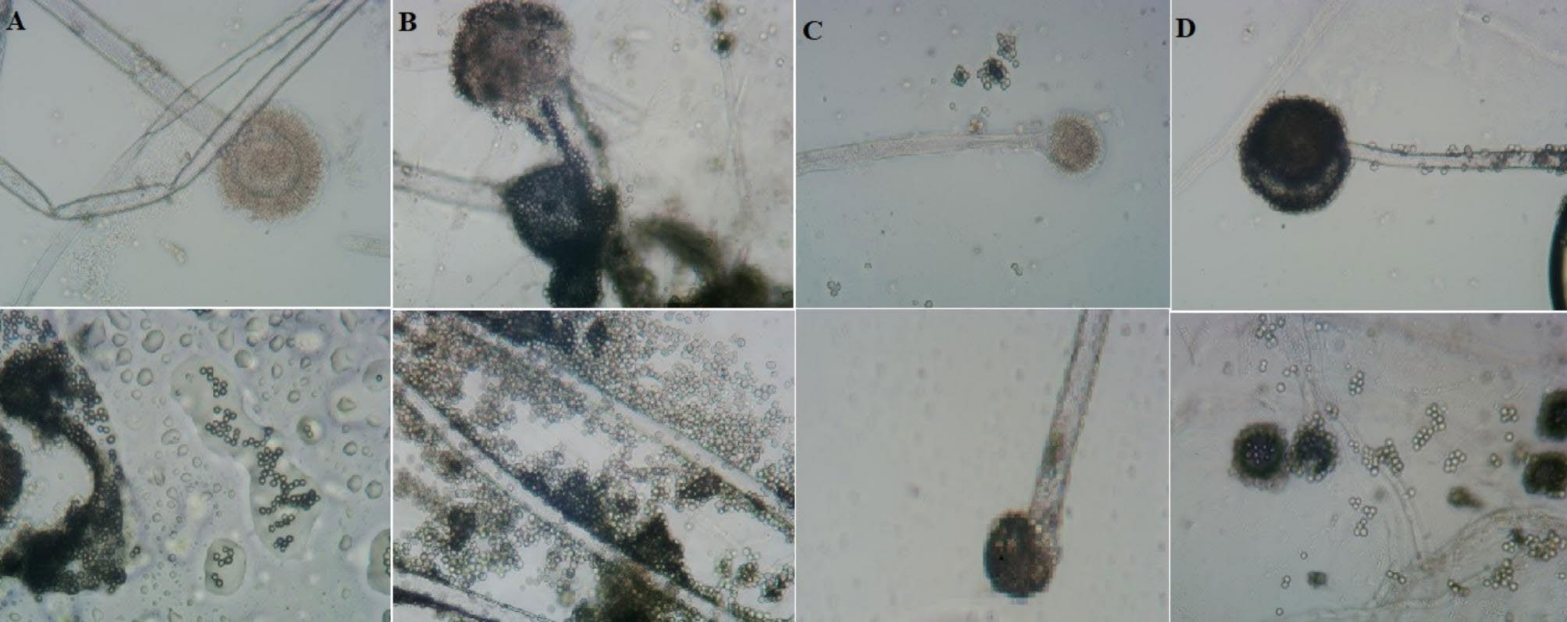
Light microscopy shows the influence of different growth media on the microscopic characteristics of *A. flavus*. **A**; the vesicle on CYA, and the shape of conidia, **B**; the vesicle on MEA, and the shape of conidia, **C**; the vesicle on PDA, and the shape of conidia, **D**; the vesicle on YES, and the shape of conidia

Regarding the sporulation, it was clear to observe the tight conidial chain formed in *A. oryzae* in which the conidiospores appeared connected to form a clear tight chain. Based on the Fig. (2), the sporulation and formation of the conidial chain are weakly affected by the media type, but the most effective medium to show the clear tight chain was MEA, and the least medium was PDA, the vesicle appeared to be well-developed and grow normally when the fungus was sub-cultured on CYA compared to the size of vesicle on the other media types. Referring to *A. flavus*, Fig. (3) exhibited that the vesicle of *A. flavus* was well developed with distinctive phialides when it was cultivated on CYA and MEA. Based on our results, the vesicle size of *A. flavus* appeared larger than its counterpart in *A. oryzae*. Cultivation media highly affected the asexual sporulation in *A. flavus* unlike what happened in *A. oryzae*. Sporulation was distinctively established in presence of MEA but it was rarely established in the presence of PDA. Conclusively, based on the culture features, CYA could differentiate easily between two analogous fungi through the color, and also, the other media involved could participate in this comparison to differentiate between these two fungi through the color of colonies and the formation of white edges. As for light microscopy, it succeeded in explaining the influence of media types on the development of the vesicle and phialides as well as the sporulation process, and the results of the light microscope did not recommend PDA to investigate *Aspergillus* spp.

Morphological identification is one of the traditional means of distinguishing the species in the *Aspergillus* section *Flavi*, nonetheless, it lacks meticulousness due to the close similitude of these species [34]. Nevertheless, the grouping of *Aspergillus* isolates into sections is necessary to facilitate examination using innovative methods for characterization such as molecular and biochemical tools [35]. The nutritional media could attain adequate requirements for fungal growth, colony development, and other macroscopic and microscopic features to have readiness for phenotypic assessment [36].

### Microscopic examination by SEM

**Fig. 4:**
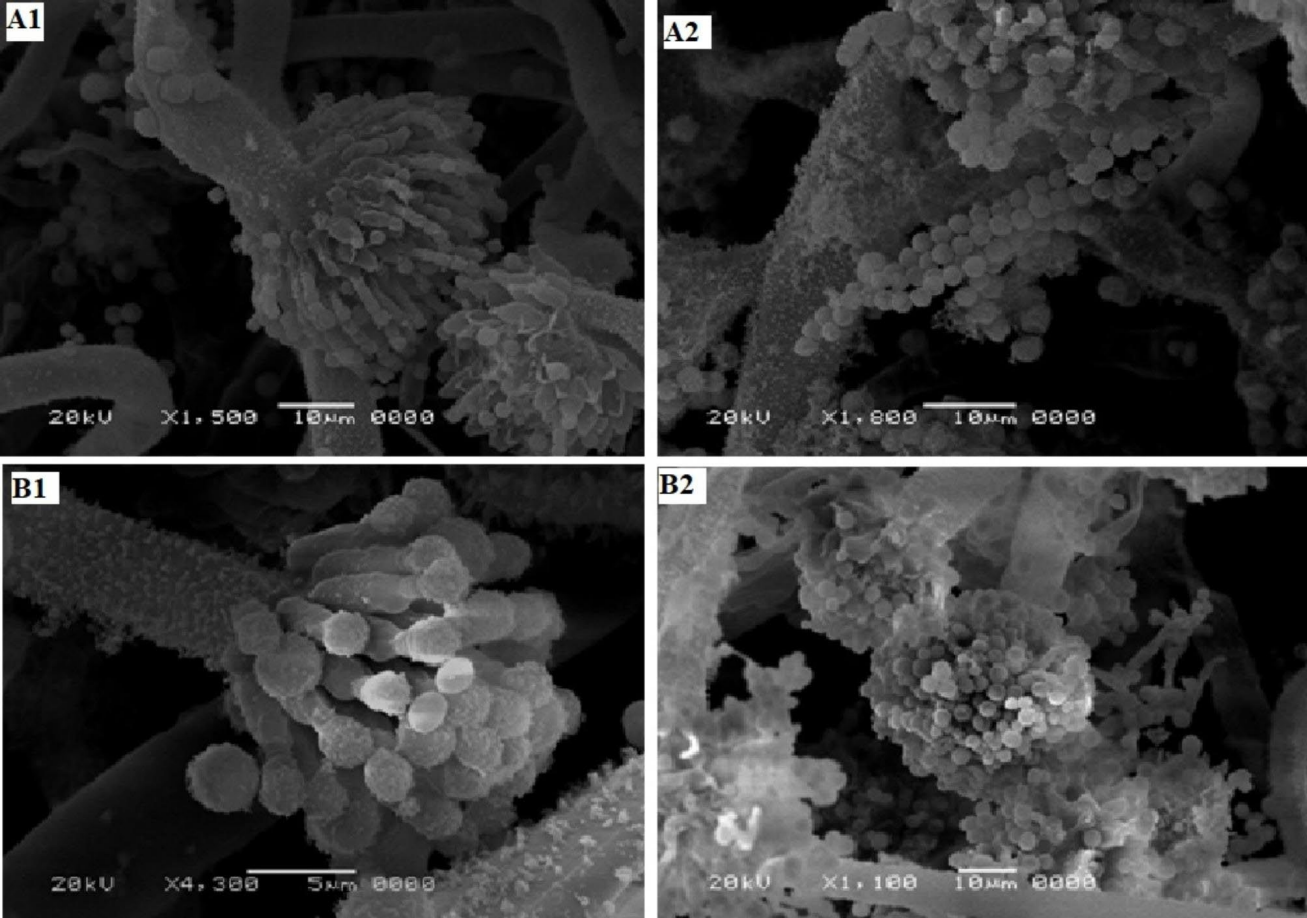
Scanning electron microscopy illustrating the pattern of both *A. oryzae*, and *A. flavus* growing on MEA. A1; the shape of vesicle and sterigmata of *A. oryzae*, A2; the obvious conidial tight chain of *A. oryzae*, B1; the small vesicle and well-organized sterigmata of *A. flavus*, B2; the conidiospores attached to the sterigmata

Regarding the examination of microscopic examination of 4 days-cultures of both *A. oryzae*, and *A. flavus*, SEM approved the same results of light microscopy by which *A. oryzae*, and its vesicle appeared bigger with intensive growing conidiospores that attached forming a tight conidial chain as shown in Fig. (4-A1 & 4-A2). On the other side, the vesicle of *A. flavus* appeared smaller in size than its counterpart in *A. oryzae*. Although the small size of the vesicle but the phialides appeared to be well developed and those phialides could be easily observed under light microscopy may be due to the scarcity of spore formation if compared with sporulation in *A. oryzae* (Fig. 4-B1 & 4-B2).

Resemblances in morphological features among both interspecific and intraspecific levels within the *Aspergilli* crucible lead to identification errors [37]. Many studies addressed scrutiny of *Aspergillus* spp. by SEM [38] to examine mycelia development, conidiophores, conidia, vesicles, and phialides [39]. Polyphasic tactics were endorsed as the ideal standards for Aspergilli classification because of the close similarity of their morphology, thus, sometimes morphology went wrong to identify strictly related species. However, noteworthy efforts recommended the use of various approaches, including phenotypic, molecular characterization, and advanced MALDI-TOF to distinguish *A. flavus* from *A. oryzae* successfully [7].

### Difference(s) in the level of ITS in both strains

According to Molecular differences based on ITS rDNA identification, the ITS segment alone does not appear to be a particularly useful gene for species differentiation of Aspergillus spp., especially between closely related species. An analysis of Beta-tubulin (*ben*A), RNA polymerase II second largest subunit (*rpb*2) and Calmodulin (*Ca*M), well-known DNA barcoding genes, on a multilocus method will yield more accurate results [40, 41]. But in this present study working on a small scale, ITS was selected as a simple, common, and most available method for a large segment of junior researchers.

**Fig. 5:**
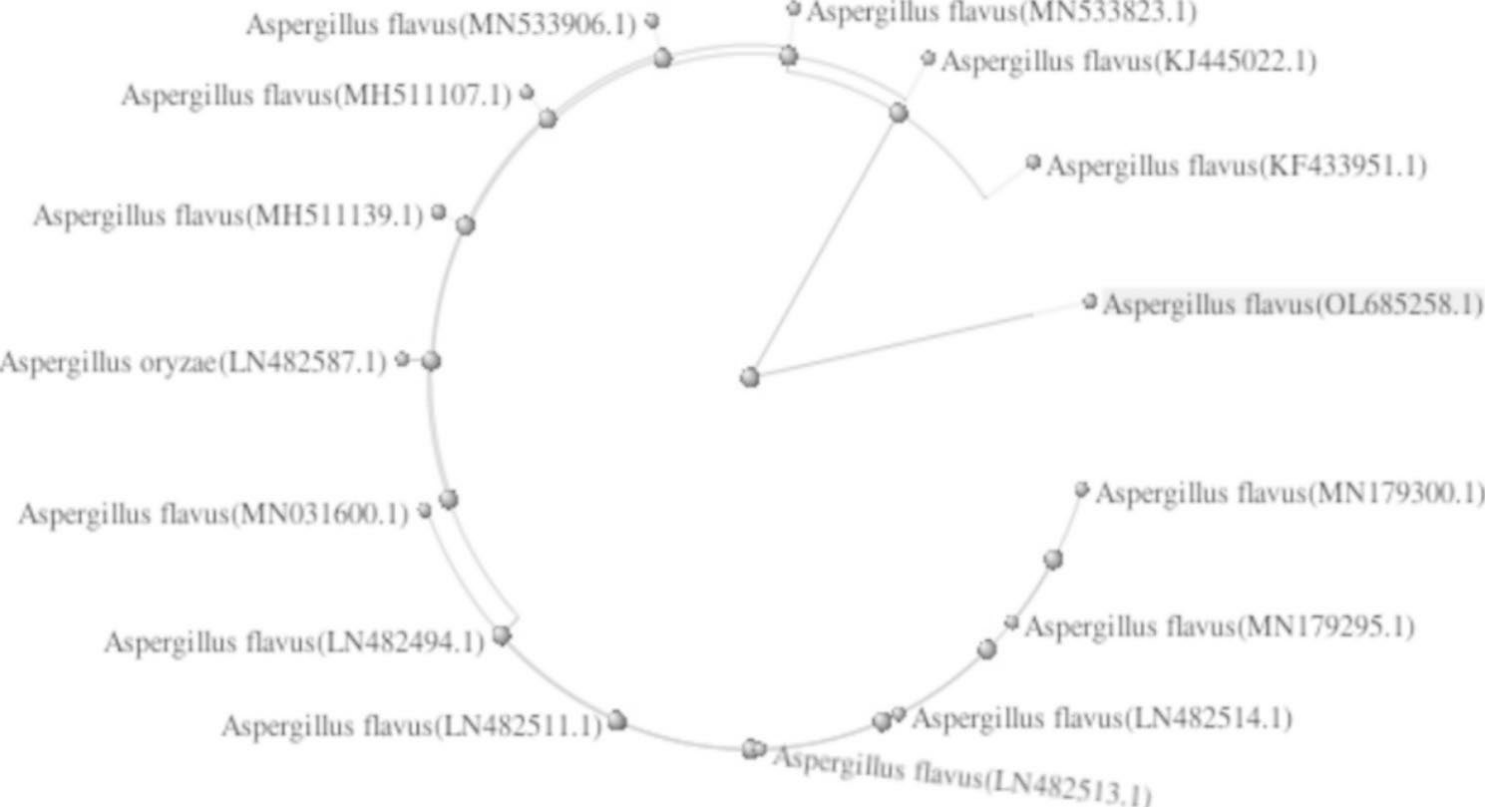
A circular cladogram shows the distance of *A. flavus* from other relative species on GenBank

**Fig. 6:**
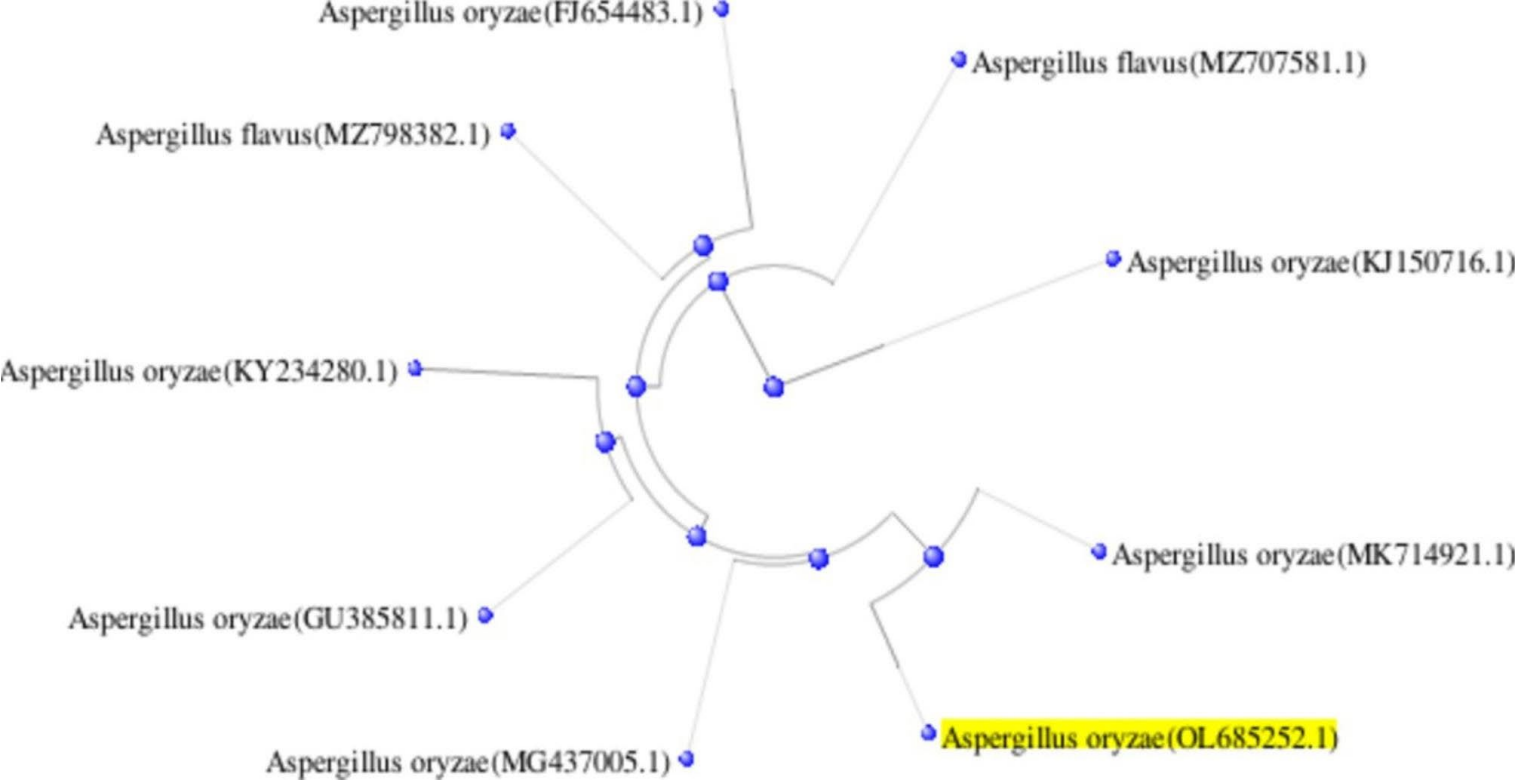
A circular cladogram shows the distance of *A. oryzae* from other relative species on GenBank

Mostly, the first step in studies related to *Aspergillus* species identification relied on the sequencing of ITS amplicons as a typical marker in filamentous fungi [42]. Amplicons of the two fungal strains were found to be 1145, and 1052 bp for *A. flavus* and *A. oryzae*, respectively. Both ITS sequences were submitted to GenBank to get accession numbers OL685258.1 for *A. flavus* (Fig. 5), and OL685252.1 for *A. oryzae* (Fig. 6). When both sequences aligned against each other by BLAST strategy, it was observed that there is a compatible fragment covered an anchor to the inquiry in a percentage of 33.8–36.7% and this fragment is identical in a percentage of 92.9–98.2%, that support our hypothesis of the high similarity of both two fungi even on the genome level.

Complete genome sequencing of both two fungi is available, providing the prospect to study somewhat genomic variances that might elucidate both fungal niches and possibly recognize virulence factors in *A. flavus*. Both *A. flavus* and *A. oryzae* are very analogous in their genome size and the number of projected genes. The assessed genome size (36·7 Mb) and projected gene number (12 097) for *A. oryzae* are similar to that of *A. flavus* (36·8 Mb and 12 179, respectively) [43]. Based on our knowledge regarding the molecular tool, the genome analyses succeeded to clarify any variation even intraspecies level variation rather than interspecies level differences. Chacón-Vargas et al. (2021) provide evidence of independent evolution of the replication of the α-amylase gene, and the genomic analyses disclose significant phenotypic and genome variation within *A. oryzae* [44].

### The contrast of their metabolic profiles by the TLC technique

Table (1) showed the presence of 12 different predicted compounds with different *Rf* values ranging from 5 to 77, it is clear to observe the abundant band detected in *A. flavus* in which 11 out of 12 bands were detected, while on the other hand, only four bands were detected in *A. oryzae*. Three out of 12 are common in both fungi; brevianamide A (*Rf* = 13), ß-nitro propionic acid (*Rf* = 31), and cyclopiazonic acid (*Rf* = 39), but there is one forecast is unique for *A. oryzae* which was kojic acid with *Rf* of 10 with brown color at long UV ate 365 nm. As for *A. flavus*, there are eight unique bands as follows; 2-pyruvoylamino benzamide (*Rf* = 5), gliotoxin (*Rf* = 20), tenuazonic acid (*Rf* = 27), aflatrem (*Rf* = 43), Aflatoxin B1 (*Rf* = 45), Aflatoxin G1 (*Rf* = 53), 3,6-dimethyl-6-hydroxyphthalide (*Rf* = 70), and itaconic acid (*Rf* = 77). Accordingly, the secondary metabolic profiles of both fungi could be easily distinguished at the first sight, toxicogenic profile of *A. flavus* is very clear in particular due to the presence of aflatoxins B1 and G1, while kojic acid is a unique component in the metabolic profile of *A. oryzae* might be the reason behind the recruitment of this fungal species in the cosmetics biotechnology. Although, kojic acid is unique for the current *A. oryzae* but sometimes *A. flavus* can do so [45].

Two marine *Aspergillus* strains were submitted to investigate their secondary metabolites profiles using HPTLC, it was observed the variation between axenic cultures and co-cultures in different ways, including some dramatical changes [46]. TLC was directed to emulate metabolic descriptions of the growing cultures in 1% and 2% glycerol. Remarkably, it showed some fluorescent compounds formed in the extracts of 2% glycerol-grown cultures relative to those produced in 1% glycerol [47]. Kojic acid is synthesized in bulky quantities by *Aspergillus oryzae* as a secondary metabolite, and it is commonly conducted in the cosmetic industry [48]. Cyclopiazonic acid, an indole-tetramic acid mycotoxin, is produced by many species of *Aspergillus* and *Penicillium* [49]. *Aspergillus* species are the dominant abundant fungi that spoil numerous foodstuffs and produce biochemicals known as mycotoxins. Aflatoxins (AFTs), aflatrem (AT), citrinin (CIT), cyclopiazonic acid (CPA), gliotoxin (GT), ochratoxin A (OTA), patulin (PAT), secalonic acids (SA), sterigmatocystin (ST) and terrein (TR), and other toxins produced by *Aspergillus* spp. that represent a major part in food and human health [50].

**Table 1:** Secondary metabolic profile of both A. flavus and A. oryzae. By THC technique

No.	*Rf*	Color (L. UV)	Forecast	*A. flavus*	*A. oryzae*
1	5	Blue	2-Pyruvoylamino benzamide	+	-
2	10	Brown	Kojic acid	-	+
3	13	Green	Brevianamide A	+	+
4	20	Yellow/Brown	Gliotoxin	+	-
5	27	Yellow/Brown	Tenuazonic acid	+	-
6	31	Violet	ß-nitropropionic acid	+	+
7	39	Violet/Brown	Cyclopiazonic acid	+	+
8	43	Yellow	Aflatrem	+	-
9	45	Green/Blue	Afla toxin B1	+	-
10	53	Violet/Brown	Afla toxin G1	+	-
11	70	Brown	3,6-Dimethyl-6-hydroxyphthalide	+	-
12	77	Violet/Brown	Itaconic acid	+	-
Total			**12**	**11**	**4**

## Conclusion

Conclusively, the current study succeeded to provide a piece of interesting information that helped to distinguish *A. flavus* from *A. oryzae* in an easy and simple method. The nutritional media tremendously contributed to this comparison by presenting significant differences in both culture color, and culture margin color. As well, light microscopy exhibited a substantial distinction in the shape of the conidial chain in which the conidiospores bonded tightly to each other to form a clear streptococcal chain in *A. oryzae*, while, conidiospores were found scattered in the case of *A. flavus*, this difference was proved by SEM too. Additionally, the molecular tool was used to identify and align both ITSs, it displayed an identity of both sequences in a percentage of 33.8–36.7% which established our hypothesis that both strains are analogous. Finally, TLC primitively showed a noticeable difference in both secondary metabolites’ profiles where the profile of *A. flavus* exhibited the presence of aflatoxins B1 and G1 as a major substantial difference. Furthermore, as a future perspective, a large group of section Flavi will be involved in order to represent a large sector of fungal isolates from different sources, countries, and habitats to maximize the benefit of this finding for the possibility to generalize this theory.

## Data Availability

All datasets generated throughout this research are included in the manuscript. Regarding ITS rDNA, the sequences were submitted to GenBank on NCBI as a member of INSDC repository. Accession numbers are available on: https://www.ncbi.nlm.nih.gov/nuccore/OL685258.1. https://www.ncbi.nlm.nih.gov/nuccore/OL685252.1.

## References

[1] Samson RA, Pitt JI. Integration of modern taxonomic methods for Penicillium and Aspergillus classification. CRC Press; 2003.

[2] Klich MA. Identification of common aspergillus species. CBS; 2002.

[3] Fischer G, Dott W (2002). Quality assurance and good laboratory practice in the mycological laboratory–compilation of basic techniques for the identification of fungi. Int J Hyg Environ Health.

[4] Kumeda Y, Asao T (2001). Heteroduplex panel analysis, a novel method for genetic identification of aspergillus section Flavi strains. Appl Environ Microbiol.

[5] Goldman GH, Osmani SA. The Aspergilli: genomics, medical aspects, biotechnology, and research methods. CRC press; 2007.

[6] Lee C-Z, Liou G-Y, Yuan G-F. Comparison of aspergillus flavus and aspergillus oryzae by amplified fragment length polymorphism. Botanical Bulletin of Academia Sinica 45; 2004.

[7] Nargesi S, Abastabar M, Valadan R, Mayahi S, Youn J-H, Hedayati MT, Seyedmousavi S (2021). Differentiation of aspergillus flavus from aspergillus oryzae targeting the cyp51A gene. Pathogens.

[8] Hedayati MT, Taghizadeh-Armaki M, Zarrinfar H, Hoseinnejad A, Ansari S, Abastabar M, Er H, Özhak B, Öğünç D, Ilkit M (2019). Discrimination of aspergillus flavus from Aspergillus oryzae by matrix‐assisted laser desorption/ionisation time‐of‐flight (MALDI‐TOF) mass spectrometry. Mycoses.

[9] Hashem AH, Suleiman WB, Abu-Elrish GM, El-Sheikh HH (2021). Consolidated Bioprocessing of Sugarcane Bagasse to Microbial Oil by newly isolated oleaginous fungus: Mortierella wolfii. Arab J Sci Eng.

[10] Hashem AH, Suleiman WB, Abu-elreesh G, Shehabeldine AM, Khalil AMA (2020). Sustainable lipid production from oleaginous fungus Syncephalastrum racemosum using synthetic and watermelon peel waste media. Bioresource Technol Rep.

[11] Hashem AH, Hasanin MS, Khalil AMA, Suleiman WB (2020). Eco-green conversion of watermelon peels to single cell oils using a unique oleaginous fungus: Lichtheimia corymbifera AH13. Waste Biomass Valoriz.

[12] Hashem AH, Abu-Elreesh G, El-Sheikh HH, Suleiman WB (2022). Isolation, identification, and statistical optimization of a psychrotolerant Mucor racemosus for sustainable lipid production. Biomass Convers Biorefinery.

[13] Gad AM, Suleiman WB, Beltagy EA, El-Sheikh H, Ibrahim HA (2021). Characterization and screening of marine-derived fungi along the coastline of Alexandria, Mediterranean Sea, Egypt. Egypt J Aquat Biology Fisheries.

[14] Gad AM, Suleiman WB, Beltagy EA, El-Sheikh H, Ibrahim HA (2021). Antimicrobial and antifouling activities of the cellulase produced by marine fungal strain; Geotrichum candidum MN638741.1. Egypt J Aquat Biology Fisheries.

[15] Gad AM, Suleiman WB, El-Sheikh HH, Elmezayen HA, Beltagy EA (2022). Characterization of cellulase from Geotrichum candidum strain Gad1 approaching bioethanol production. Arab J Sci Eng.

[16] Hendy MH, Hashem AH, Suleiman WB, Sultan MH, Abdelraof M (2023). Purification, characterization and anticancer activity of L-methionine γ-lyase from thermo-tolerant aspergillus fumigatus. Microb Cell Fact.

[17] Koczorski P, Furtado BU, Gołębiewski M, Hulisz P, Baum C, Weih M, Hrynkiewicz K. (2021) The effects of host plant genotype and environmental conditions on fungal community composition and phosphorus solubilization in willow short rotation coppice. Frontiers in Plant Science 12 10.3389/fpls.2021.647709 PMC8287252 34290719

[18] Suleiman WB, Helal EE (2022). Chemical constituents and potential pleiotropic activities of Foeniculum vulgare (fennel) ethanolic extract; in vitro approach egyptian. J Chem.

[19] El-Naggar HA, Bashar MA, Rady I, El-Wetidy MS, Suleiman WB, Al-Otibi FO, Al-Rashed SA, El-Maoula A, Lamiaa M, Salem E-SS (2022). Two Red Sea Sponge extracts (Negombata magnifica and Callyspongia siphonella) Induced Anticancer and Antimicrobial Activity. Appl Sci.

[20] Shawky M, Suleiman WB, Farrag AA (2021). Antibacterial Resistance Pattern in Clinical and non-clinical Bacteria by phenotypic and genotypic Assessment. J Pure Appl Microbiol.

[21] Soliman MO, Suleiman WB, Roushdy MM, Elbatrawy EN, Gad AM (2022). Characterization of some bacterial strains isolated from the egyptian Eastern and Northern coastlines with antimicrobial activity of Bacillus zhangzhouensis OMER4. Acta Oceanol Sin.

[22] Kamel A, Suleiman WB, Elfeky A, El-Sherbiny GM, Elhaw M (2022). Characterization of bee venom and its synergistic effect combating antibiotic resistance of Pseudomonas aeruginosa. Egypt J Chem.

[23] Suleiman WB (2020). In vitro estimation of superfluid critical extracts of some plants for their antimicrobial potential, phytochemistry, and GC–MS analyses. Ann Clin Microbiol Antimicrob.

[24] El-Naggar HA (2022). Mosquitocidal activity of Ophiocoma scolopendrina extracts against Culex pipiens and their antimicrobial potential. Egypt J Aquat Biology Fisheries.

[25] Suleiman WB, El Bous M, Ibrahim M, El Baz H (2019). In vitro evaluation of Syzygium aromaticum L. ethanol extract as biocontrol agent against postharvest tomato and potato diseases. Egypt J Bot.

[26] Klich MA. (2002) Identification of common Aspergillus species. Centraalbureau voor schimmelcultures

[27] Abdel-Razek A, El-Sheikh H, Suleiman W, Taha TH, Mohamed M (2020). Bioelimination of phenanthrene using degrading bacteria isolated from petroleum soil: safe approach. Desalination Water Treat.

[28] Ali OM, Hasanin MS, Suleiman WB, Helal EE, Hashem AH (2022). Green biosynthesis of titanium dioxide quantum dots using watermelon peel waste: antimicrobial, antioxidant, and anticancer activities. Biomass Convers Biorefinery.

[29] White TJ, Bruns T, Lee S, Taylor J (1990). Amplification and direct sequencing of fungal ribosomal RNA genes for phylogenetics. PCR protocols: a guide to methods and applications.

[30] Paterson R, Bridge P. Biochemical techniques for filamentous fungi. Volume 1. CaB International; 1994.

[31] Suleiman WB, Shehata RM, Younis A (2022). In vitro assessment of multipotential therapeutic importance of Hericium erinaceus mushroom extracts using different solvents. Bioresources and Bioprocessing.

[32] Khalil AMA, Hashem AH (2018). Morphological changes of conidiogenesis in two aspergillus species. J Pure Appl Microbiol.

[33] Sharma G (2010). Influence of culture media on growth, colony character and sporulation of fungi isolated from decaying vegetable wastes. J yeast fungal Res.

[34] Norlia M, Jinap S, Nor-Khaizura M, Son R, Chin C (2018). Polyphasic approach to the identification and characterization of aflatoxigenic strains of aspergillus section Flavi isolated from peanuts and peanut-based products marketed in Malaysia. Int J Food Microbiol.

[35] Zulkifli NA, Zakaria L (2017). Morphological and molecular diversity of aspergillus from corn grain used as livestock feed. HAYATI J Biosci.

[36] Thathana MG, Murage H, Abia ALK, Pillay M (2017). Morphological characterization and determination of aflatoxin-production potentials of aspergillus flavus isolated from maize and soil in Kenya. Agriculture.

[37] Okayo RO, Andika DO, Dida MM, K’Otuto GO, Gichimu BM. (2020) Morphological and molecular characterization of toxigenic Aspergillus flavus from groundnut kernels in Kenya. International Journal of Microbiology 2020 10.1155/2020/8854718 PMC7492892 32963542

[38] Shakeel Q, Lyu A, Zhang J, Wu M, Li G, Hsiang T, Yang L (2018). Biocontrol of Aspergillus flavus on peanut kernels using Streptomyces yanglinensis 3–10. Front Microbiol.

[39] El-Kadi SM, El-Fadaly HA, El-Gayar E-SM (2018). Scanning Electron Microscopy of Fungi isolated from some cake samples. Int J Microbiol Application.

[40] Krimitzas A, Pyrri I, Kouvelis VN, Kapsanaki-Gotsi E, Typas MA. (2013) A phylogenetic analysis of Greek isolates of Aspergillus species based on morphology and nuclear and mitochondrial gene sequences. BioMed research international 2013 10.1155/2013/260395 PMC3665174 23762830

[41] Visagie CM, Houbraken J (2020). Updating the taxonomy of aspergillus in South Africa. Stud Mycol.

[42] Makhlouf J, Carvajal-Campos A, Querin A, Tadrist S, Puel O, Lorber S, Oswald IP, Hamze M, Bailly J-D, Bailly S (2019). Morphologic, molecular and metabolic characterization of aspergillus section Flavi in spices marketed in Lebanon. Sci Rep.

[43] Payne G, Nierman W, Wortman J, Pritchard B, Brown D, Dean R, Bhatnagar D, Cleveland T, Machida M, Yu J (2006). Whole genome comparison of aspergillus flavus and A. oryzae. Med Mycol.

[44] Chacón-Vargas K, McCarthy CO, Choi D, Wang L, Yu J-H, Gibbons JG. (2021) Comparison of Two Aspergillus oryzae Genomes From Different Clades Reveals Independent Evolution of Alpha-Amylase Duplication, Variation in Secondary Metabolism Genes, and Differences in Primary Metabolism. Frontiers in microbiology:1958 10.3389/fmicb.2021.691296 PMC8313989 34326825

[45] Ola AR, Metboki G, Lay CS, Sugi Y, Rozari PD, Darmakusuma D, Hakim EH (2019). Single production of kojic acid by aspergillus flavus and the revision of flufuran. Molecules.

[46] Wang Y, Glukhov E, He Y, Liu Y, Zhou L, Ma X, Hu X, Hong P, Gerwick WH, Zhang Y (2022). Secondary metabolite variation and bioactivities of two Marine aspergillus strains in static co-culture investigated by Molecular Network Analysis and multiple database mining based on LC-PDA-MS/MS. Antibiotics.

[47] Machushynets NV, Wu C, Elsayed SS, Hankemeier T, van Wezel GP (2019). Discovery of novel glycerolated quinazolinones from Streptomyces sp. MBT27. J Ind Microbiol Biotechnol.

[48] Terabayashi Y, Sano M, Yamane N, Marui J, Tamano K, Sagara J, Dohmoto M, Oda K, Ohshima E, Tachibana K (2010). Identification and characterization of genes responsible for biosynthesis of kojic acid, an industrially important compound from aspergillus oryzae. Fungal Genet Biol.

[49] Chang P-K, Horn BW, Dorner JW (2009). Clustered genes involved in cyclopiazonic acid production are next to the aflatoxin biosynthesis gene cluster in aspergillus flavus. Fungal Genet Biol.

[50] Navale V, Vamkudoth KR, Ajmera S, Dhuri V (2021). Aspergillus derived mycotoxins in food and the environment: prevalence, detection, and toxicity. Toxicol Rep.

